# Age has a U-shaped relationship with breast cancer outcomes in women: a cohort study

**DOI:** 10.3389/fonc.2023.1265304

**Published:** 2023-10-04

**Authors:** Yujie Xie, Yongqing Deng, Suosu Wei, Zhen Huang, Lihui Li, Kai Huang, Chunyu Wei, Jinan Xu, Lingguang Dong, Qiuhuan Zhang, Jiehua Zhao, Quanqing Zou, Jianrong Yang

**Affiliations:** ^1^ Department of Breast and Thyroid Surgery, People’s Hospital of Guangxi Zhuang Autonomous Region, Nanning, Guangxi, China; ^2^ The Family Planning Office of Guangxi Academy of Medical Sciences and the People's Hospital of Guangxi Zhuang Autonomous Region, Nanning, Guangxi, China; ^3^ Department of Scientific Cooperation of Guangxi Academy of Medical Sciences, People’s Hospital of Guangxi Zhuang Autonomous Region, Nanning, Guangxi, China; ^4^ Department of Colorectal and Anal Surgery, People’s Hospital of Guangxi Zhuang Autonomous Region, Nanning, Guangxi, China

**Keywords:** breast cancer, overall survival, breast cancer-specific survival, disease-free survival, prognosis, age

## Abstract

**Background and Objectives:**

Age is a significant determinant of susceptibility to breast cancer. Currently, the available evidence regarding the non-linear correlation between the age of diagnosis and the prognosis of breast cancer patients is contradictory. Insufficient data currently exist regarding the influence of age at diagnosis on the prognosis of breast cancer. The objective of our investigation was to examine the relationship between age at diagnosis and overall survival (OS), breast cancer-specific survival (BCSS), and disease-free survival (DFS).

**Methods:**

This retrospective cohort study included 1054 patients diagnosed with breast cancer between March 7, 2013 and December 31, 2019. The hazard ratios (HRs) and 95% confidence interval (CI) for OS, BCSS, DFS were assessed using Cox proportional hazard ratio models and restricted cubic splines (RCS).

**Results:**

The study included 1054 breast cancer patients who met the criteria. With a median follow-up of 4.86 years, 71 patients (6.74%) died and 144 patients (13.66%) relapsed. After multivariable adjustment, age showed a U-shaped association with OS, BCSS, and DFS, with significantly higher risk at two ends, with age inflection points of 44, 44, and 41 years for OS, BCSS, and DFS, respectively. For OS, Quartile 1 (HR, 2.09; 95% CI: 0.90-4.84), Quartile 3 (HR, 2.44; 95% CI: 1.05-5.65) and Quartile 4 (HR, 3.38; 95% CI: 1.51-7.54) had poorer OS compared with Quartile 2. Similar results were found for BCSS and DFS.

**Conclusions:**

This study confirmed a U-shaped association between age at diagnosis and breast cancer outcome.

## Background

Breast cancer is currently the most prevalent form of cancer among women, with an estimated 2.26 million diagnoses and 685,000 deaths in 2020 (1). The projected global cancer burden is anticipated to increase by 50% in 2040 as compared to 2020 (2).

Breast cancer is relatively common in middle-aged and elderly women. In China, about 30% of breast cancers are diagnosed in people over the age of 60, a proportion that exceeds 50% abroad (3, 4). Women aged 50 and above constitute roughly 82% of new breast cancer diagnoses (4). Conversely, breast cancer incidence in patients under the age of 40 and 30 accounts for only 6.5% and 0.6%, respectively (5). The above research shows that there may be a certain relationship between age and the occurrence of breast cancer.

The influence of age on the prognosis of breast cancer has been examined, yet there exists contradictory evidence regarding this matter (6–8). One study showed that older patients had a poorer prognosis than younger patients (9). In postmenopausal elderly breast cancer patients, Research has demonstrated that an increased age at diagnosis is associated with a unfavorable prognosis (6, 10). Nevertheless, surgical intervention has been found to enhance both overall survival (OS) and breast cancer-specific survival (BCSS) in elderly patients, thereby establishing it as an efficacious and secure treatment approach (11). In contrast to the prevailing notion that older patients experience a more adverse prognosis compared to their younger counterparts, certain studies have revealed that younger patients exhibit inferior survival outcomes in comparison to older patients (12–15). The 75-month OS incidence (78%) and DFS (62%) were significantly worse (p < 0.05) in women aged 35 years and younger compared to OS (89%) and disease-free survival (DFS) (78%) in women aged 36-45 years (16). It is worth noting that younger patients exhibited larger tumors and a higher likelihood of developing axillary lymph node metastases in comparison to older patients (17, 18). This discrepancy may contribute to the comparatively poorer survival outcomes observed in younger patients. Consequently, it is imperative to explore the correlation between age at diagnosis and survival among individuals with cancer.

## Materials and methods

### Data source and study population

**Figure 1:**
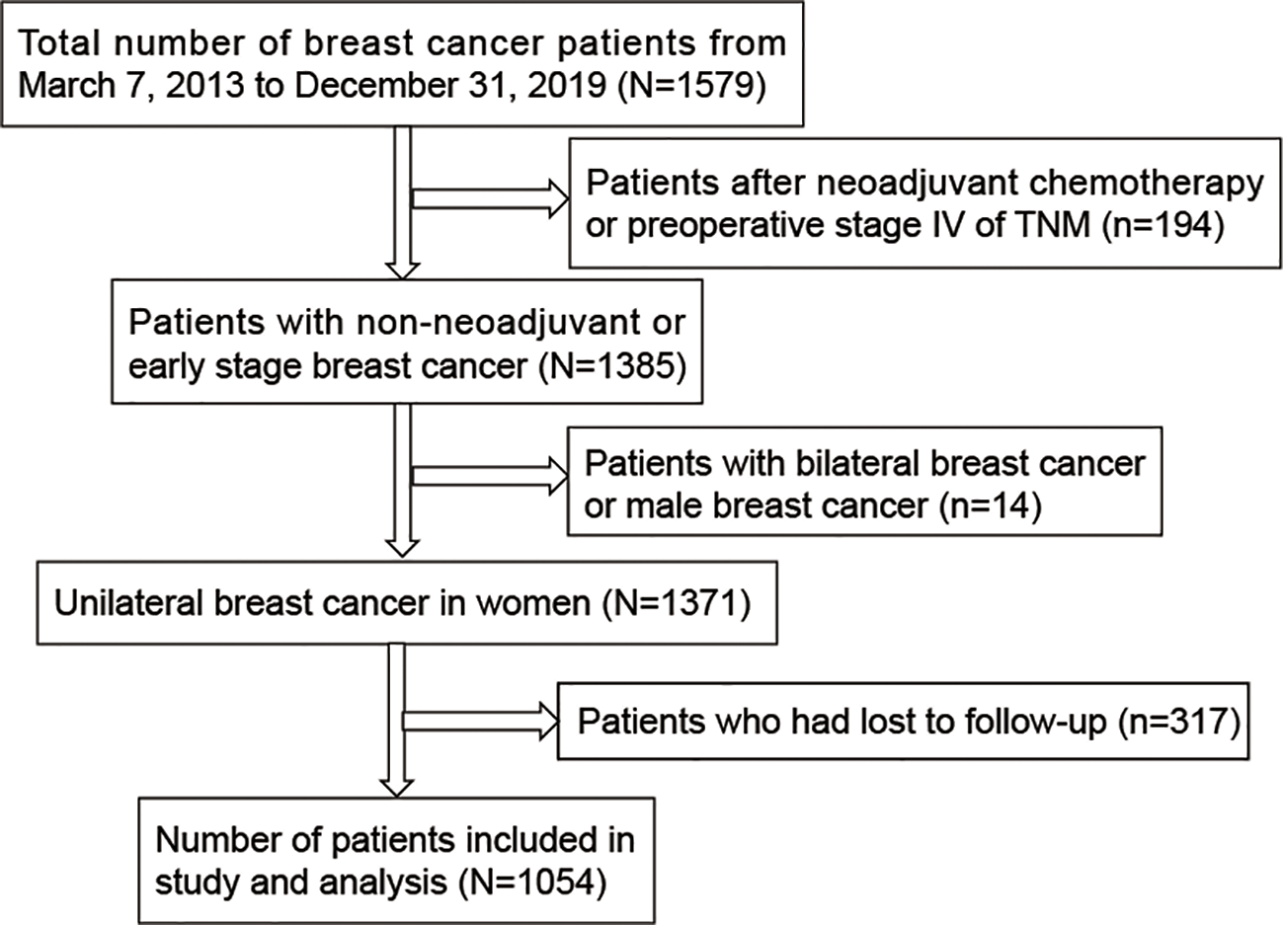
Flow chart of study selection process.

The current historical cohort study included all inpatients diagnosed with breast cancer at the People’s Hospital of Guangxi Zhuang Autonomous Region, China, from March 7, 2013 to December 31, 2019 (registration site http://www.chictr.org.cn/index.aspx; registration number ChiCTR2200058542). In the present study 1579 patients were selected. Of these, only 1054 breast cancers were included. Patients based on the following criteria were excluded: breast cancer patients who had neoadjuvant chemotherapy, preoperative stage IV, bilateral breast cancer, male breast cancer and lost to follow-up. The inclusion and exclusion criteria for the enrolled patients were shown in **Figure 1**. The study protocol conformed to the ethical guidelines of the Declaration of Helsinki (6th revision, 2008) and was approved by the Ethics Committees of the People’s Hospital of Guangxi Zhuang Autonomous Region, China. Individual informed consent was not obtained in this study because we analyzed anonymized electronic medical records data as aggregates, with no individual health data available.

### Construction of variables

This study focused on the age at diagnosis of breast cancer as the primary independent variable, stratified as Quartile 1 (Q1: age 22–43 years), Quartile 2 (Q2: 44 to 50 years), Quartile 3 (Q3: 51-58 years) and Quartile 4 (Q4: 59-90 years) by quartiles. That is, all breast cancer patients are listed in order of age, and we divided the total data into four equal parts, with each quartile containing 25% of breast cancer patients. Clinical characteristics included age at diagnosis, T category, N category, stage of TNM, tumor size, histopathological grade, lymph nodes in the axilla status (LN), molecular subtype, nuclear-associated antigen ki-67 (Ki-67) level, Status of both ER and PR hormone receptors, HER2 status, operation type, adjuvant therapy, hormonal therapy and follow-up time. Of which, TNM stage is divided into 0 (TisN0M0), 1 (T1N0M0), 2 (T1N1M0, T2N0-1M0 and T3N0M0), 3 (T1-2N2M0, T3N1-2M0, T4Nany M0 and Tany N3M0) and Unknown (unable to determine or sample size is missing). Operation type is divided into BCS (breast-conserving surgery) and M (mastectomy). With reference to the Chinese Anti-Cancer Society Breast Cancer Diagnosis and Treatment Guidelines and Specifications (2020) (19) and the Guidelines for HER-2 Detection in Breast Cancer (2019) (20), in our study the tumors were classified into Luminal A, Luminal B (HER-2 -), HER-2 + (HR* +), HER-2 + (HR* -) and triple negative subtypes.

### Follow-up and endpoints

The follow-up period was determined by the initial diagnosis date of breast cancer, and patients were monitored until June 30, 2022 or until their demise. Treatment specifics and pertinent data were obtained from electronic medical records or telephone follow-up. The study endpoints encompassed OS, BCSS and DFS. OS referred to the time elapsed between diagnosis of breast cancer and death caused by any cause. BCSS measured the total length of time between diagnosis and death from breast cancer-related causes. DFS was defined as the first breast cancer recurrence at any site after the surgery date. Patients without events were censored at the time of their last follow-up.

### Statistical analysis

Categorical or continuous variables are presented as median and interquartile range (IQR) or frequency (percentage) base on the data of baseline characteristics, respectively. In order to compare the baseline characteristics of the groups, chi-square tests for categorical variables and Wilcoxon rank sum tests for continuous variables were used. Multivariable Cox regression was used to assess the effect of age on OS, BCSS and DFS to adjust for the effects of independent variables (tumor size, molecular subtype, lymph nodes in the axilla, operation type, adjuvant therapy, hormonal therapy). Covariate selection and other potential confounders were identified using a backward selection procedure, clinical significance and literature data, respectively. The crude and adjusted (HR) and 95% (CI) were calculated for the risk for OS, BCSS and DFS according to age group. By modeling age group with restricted cubic splines (RCS) and adjusting for potential confounders, a possible nonlinear association was identified. Smooth curve fitting was used first to determine if the independent variable is interval-based. The intervals were fitted with segmented regression using separate line segments (also called piece-wise regression). The log-likelihood ratio test for nonlinearity of smooth curve fitting was conducted by comparing the non-segmented model with a segmented regression model to calculate the P value. The threshold level of age was determined when a maximum likelihood model was found at the inflection point. We used R 3.4.3 (http://www.R-project.org, The R Foundation) for all analyses. P values less than 0.05 were considered statistically significant.

## Results

### Demographic and clinical characteristics of patients

The current historical cohort study included all inpatients diagnosed with breast cancer at the People’s Hospital of Guangxi Zhuang Autonomous Region, China, from March 7, 2013 to December 31, 2019. In the present study 1579 patients were selected. Of these, the study included 1054 breast cancer patients who met the criteria. There was a median age of 51.00 years (interquartile range, 44.00-59.00) at diagnosis. The baseline clinicopathologic characteristics of those selected according to their age quartile are shown in **Table 1**. The definition of “higher age quartile” is Q3(51-58y)-Q4(59-90y). Significant differences were observed in Ki-67 level, PR status, operation type, and adjuvant therapy by age quartile. For the higher age quartile, patients had a significantly higher PR-negative tumors and mastectomy rate. Q3 and Q4 had higher PR-negative tumors, Q3 had the highest (42.8%) *vs* Q4 (40.34%). The proportion of mastectomy was higher in Q3 and Q4, with the highest number of mastectomy in Q4 (90.34%) *vs* Q3 (83.76%). In contrast, Ki-67 levels, breast conserving surgery, and adjuvant chemotherapy were significantly lower in the higher age quartile (all P < 0.05). The proportion of tumors with high Ki-67 expression (Ki-67 > 30%) was lower in Q3 and Q4, with Q4 having the lowest rate of tumors with high Ki-67 expression (21.72%) compared to Ki-67 in Q3 (30.63%). The percentage of breast-conserving surgeries was lower in Q3 and Q4, where it was lowest in Q4 (9.66%) compared to Q3 (16.24%). The proportion of patients treated with adjuvant chemotherapy was lower in Q3 and Q4, with Q4 having the lowest proportion of adjuvant chemotherapy (35.86%) compared to Q3 (47.23%). Unlike the previous indicators, radiation therapy is U-shaped in all age groups, with the lowest in Q3 (2.58%) and the highest in Q4 (4.14%). Compared with Q2 (2.86%), Q1 (3.23%) has a higher radiation therapy rate.

**Table 1:** Comparison of treatments and clinical and pathological characteristics of tumors according to age at breast cancer diagnosis.

Variables	Total	Q1(22-43years)	Q2(44-50years)	Q3(51-58years)	Q4(59-90years)	P-value
**Number of patients**	1054	248	245	271	290	
**AGE (years)**	51.00 (44.00-59.00)	39.00 (36.00-41.00)	47.00 (45.00-49.00)	54.00 (52.00-56.00)	64.50 (61.00-70.00)	**<0.001**
**T category [n (%)]**						0.149
Tis	22 (2.09%)	7 (2.82%)	5 (2.04%)	3 (1.11%)	7 (2.41%)	
T1	452 (42.88%)	98 (39.52%)	107 (43.67%)	118 (43.54%)	129 (44.48%)	
T2	467 (44.31%)	105 (42.34%)	105 (42.86%)	124 (45.76%)	133 (45.86%)	
T3	62 (5.88%)	18 (7.26%)	14 (5.71%)	19 (7.01%)	11 (3.79%)	
T4	2 (0.19%)	0 (0.00%)	0 (0.00%)	1 (0.37%)	1 (0.34%)	
Unknown	49 (4.65%)	20 (8.06%)	14 (5.71%)	6 (2.21%)	9 (3.10%)	
**N category [n (%)]**						0.290
N0	592 (56.17%)	133 (53.63%)	133 (54.29%)	158 (58.30%)	168 (57.93%)	
N1	257 (24.38%)	64 (25.81%)	57 (23.27%)	64 (23.62%)	72 (24.83%)	
N2	119 (11.29%)	33 (13.31%)	36 (14.69%)	27 (9.96%)	23 (7.93%)	
N3	66 (6.26%)	12 (4.84%)	17 (6.94%)	19 (7.01%)	18 (6.21%)	
Unknown	20 (1.90%)	6 (2.42%)	2 (0.82%)	3 (1.11%)	9 (3.10%)	
**Stage of TNM**						0.541
0	25 (2.37%)	8 (3.23%)	7 (2.86%)	3 (1.11%)	7 (2.41%)	
1	280 (26.57%)	58 (23.39%)	64 (26.12%)	74 (27.31%)	84 (28.97%)	
2	482 (45.73%)	116 (46.77%)	103 (42.04%)	125 (46.13%)	138 (47.59%)	
3	206 (19.54%)	49 (19.76%)	53 (21.63%)	57 (21.03%)	47 (16.21%)	
Unknown	61 (5.79%)	17 (6.85%)	18 (7.35%)	12 (4.43%)	14 (4.83%)	
**Tumor size(cm)**						0.159
<2cm	463 (43.93%)	101 (40.73%)	111 (45.31%)	119 (43.91%)	132 (45.52%)	
>2cm	538 (51.04%)	127 (51.21%)	120 (48.98%)	144 (53.14%)	147 (50.69%)	
Unknown	53 (5.03%)	20 (8.06%)	14 (5.71%)	8 (2.95%)	11 (3.79%)	
**Histopathological grade**						0.163
Low intermediate (G1, G2)	617 (58.54%)	132 (53.23%)	149 (60.82%)	160 (59.04%)	176 (60.69%)	
High, G3	258 (24.48%)	72 (29.03%)	54 (22.04%)	73 (26.94%)	59 (20.34%)	
Unknown	179 (16.98%)	44 (17.74%)	42 (17.14%)	38 (14.02%)	55 (18.97%)	
**LN N (%)**						0.695
Negative	591 (56.07%)	134 (54.03%)	134 (54.69%)	154 (56.83%)	169 (58.28%)	
Positive	439 (41.65%)	108 (43.55%)	108 (44.08%)	111 (40.96%)	112 (38.62%)	
Unknown	24 (2.28%)	6 (2.42%)	3 (1.22%)	6 (2.21%)	9 (3.10%)	
**Molecular subtype**						0.165
Luminal A	256 (24.29%)	55 (22.18%)	61 (24.90%)	56 (20.66%)	84 (28.97%)	
Luminal B (HER-2-)	374 (35.48%)	84 (33.87%)	93 (37.96%)	100 (36.90%)	97 (33.45%)	
HER-2+ (HR*+)	122 (11.57%)	32 (12.90%)	28 (11.43%)	40 (14.76%)	22 (7.59%)	
HER-2+ (HR*-)	133 (12.62%)	27 (10.89%)	29 (11.84%)	40 (14.76%)	37 (12.76%)	
Triple negative	120 (11.39%)	36 (14.52%)	22 (8.98%)	25 (9.23%)	37 (12.76%)	
Unknown	49 (4.65%)	14 (5.65%)	12 (4.90%)	10 (3.69%)	13 (4.48%)	
**Ki-67 level**						**0.002**
<14%	294 (27.89%)	65 (26.21%)	62 (25.31%)	67 (24.72%)	100 (34.48%)	
14%≤1 ≤ 30%	402 (38.14%)	81 (32.66%)	91 (37.14%)	116 (42.80%)	114 (39.31%)	
>30%	311 (29.51%)	87 (35.08%)	78 (31.84%)	83 (30.63%)	63 (21.72%)	
Unknown	47 (4.46%)	15 (6.05%)	14 (5.71%)	5 (1.85%)	13 (4.48%)	
**ER status**						0.055
Negative	289 (27.42%)	67 (27.02%)	56 (22.86%)	81 (29.89%)	85 (29.31%)	
Positive	753 (71.44%)	174 (70.16%)	188 (76.73%)	188 (69.37%)	203 (70.00%)	
Unknown	12 (1.14%)	7 (2.82%)	1 (0.41%)	2 (0.74%)	2 (0.69%)	
**PR status**						**0.010**
Negative	396 (37.57%)	87 (35.08%)	76 (31.02%)	116 (42.80%)	117 (40.34%)	
Positive	643 (61.01%)	153 (61.69%)	167 (68.16%)	152 (56.09%)	171 (58.97%)	
Unknown	15 (1.42%)	8 (3.23%)	2 (0.82%)	3 (1.11%)	2 (0.69%)	
**HER-2 status**						0.188
Negative	758 (71.92%)	179 (72.18%)	179 (73.06%)	178 (65.68%)	222 (76.55%)	
Positive	252 (23.91%)	58 (23.39%)	57 (23.27%)	80 (29.52%)	57 (19.66%)	
Unknown	44 (4.17%)	11 (4.44%)	9 (3.67%)	13 (4.80%)	11 (3.79%)	
**Operation type**						**0.001**
BCS	170 (16.13%)	56 (22.58%)	42 (17.14%)	44 (16.24%)	28 (9.66%)	
M	884 (83.87%)	192 (77.42%)	203 (82.86%)	227 (83.76%)	262 (90.34%)	
**Adjuvant therapy**						**0.002**
Chemotherapy	476 (45.16%)	121 (48.79%)	123 (50.20%)	128 (47.23%)	104 (35.86%)	
Radiotherapy	34 (3.23%)	8 (3.23%)	7 (2.86%)	7 (2.58%)	12 (4.14%)	
Both	262 (24.86%)	61 (24.60%)	63 (25.71%)	71 (26.20%)	67 (23.10%)	
None	282 (26.76%)	58 (23.39%)	52 (21.22%)	65 (23.99%)	107 (36.90%)	
**Hormonal therapy N (%)**						0.254
No	419 (39.75%)	100 (40.32%)	89 (36.33%)	102 (37.64%)	128 (44.14%)	
Yes	635 (60.25%)	148 (59.68%)	156 (63.67%)	169 (62.36%)	162 (55.86%)	
**Follow-up time (years)**	4.86(3.26-6.83)	4.90(3.42-6.86)	5.01 (3.63-7.10)	4.53 (3.06-6.80)	4.85 (3.19-6.55)	0.115

median and Q1-Q3 for continuous variables; Number (%) for categorical variables; T, tumor size; N, lymph nodes; ER, estrogen receptor; PR: progesterone receptor; HER-2, human epidermal growth factor receptor 2; BCS, breast-conserving surgery; M, mastectomy; LN, lymph nodes in the axilla status.

The bold values denote statistical significance at P<0.05 level.

### Non-linear association of age with OS, BCSS and DFS

**Figure 2:**
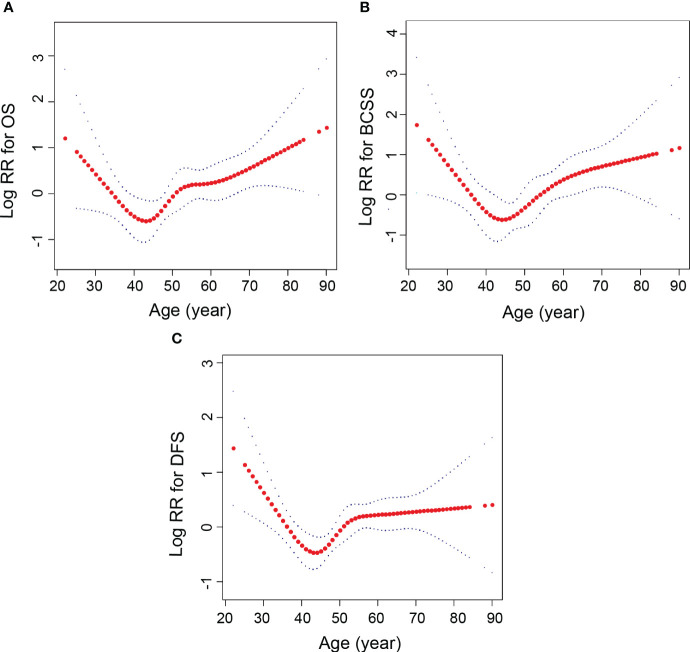
Relationships between age and the probability of OS, BCSS and DFS. **(A)** OS, **(B)** BCSS, **(C)** DFS. Non-linear associations between age and OS, BCSS and DFS were found (P < 0.05). The y-axis of Log RR is defined as the logarithm of the relative risk. Each red dot represents an age. The red line represents the age and the blue line represents its corresponding 95% confidence interval. Adjusted for tumor size, molecular subtype, lymph nodes in the axilla status, operation type, adjuvant therapy, hormonal therapy.

Adjusted smooth curve fitting revealed a U-shaped relationship between age group and OS, BCSS, and DFS (**Figure 2**). As a result of threshold effect analysis (**Table 2**), age was associated with OS, BCSS, and DFS in a significant U-shaped relationship (P for log likelihood ratio test <0.05). OS, BCSS and DFS identified age inflection points of 44, 44 and 41 years, respectively. On the left of inflection point, the risk for OS (fully adjusted (HR), 0.94; 95% (CI), 0.88, 1.0), BCSS (fully adjusted (HR), 0.77; 95% (CI), 0.66, 0.89) and DFS (fully adjusted (HR), 0.92; 95% (CI), 0.86, 0.98) were negatively associated with age. Conversely, on the right of inflection point, the risk for OS (fully adjusted [HR], 1.05; 95% [CI], 1.02, 1.08), BCSS (fully adjusted [HR], 1.04; 95% [CI], 1.01, 1.07) and DFS (fully adjusted [HR], 1.02; 95% [CI], 1.01, 1.04) was positively associated with age.

**Table 2:** Threshold effect analysis of age on OS, BCSS, and DFS among 1054 breast patients.

Variable	Model 1	P-value	Model 2	P-value	Model 3	P-value
	HR (95% CI) *		HR (95% CI) *		HR (95% CI) *	
OS						
Age, years☆	1.02(1.00-1.04)	0.055	1.02(1.00-1.04)	**0.031**	1.03(1.01-1.05)	**0.021**
Inflection point★						
Age<44 years	0.93 (0.87, 1.00)	**0.039**	0.94 (0.88, 1.01)	0.073	0.94 (0.88, 1.01)	0.074
Age>44 years	1.04 (1.01, 1.06)	**0.002**	1.04 (1.01, 1.06)	**0.002**	1.05 (1.02, 1.08)	**0.001**
P for log likelihood ratio test		**0.015**		**0.025**		**0.017**
BCSS						
Age, years☆	1.02(1.00-1.05)	0.103	1.02(1.00-1.05)	0.057	1.02(1.00-1.05)	0.063
Inflection point★						
Age<44 years	0.90 (0.84, 0.97)	**0.007**	0.93 (0.85, 1.01)	0.100	0.93 (0.85, 1.02)	0.119
Age>44 years	1.05 (1.02, 1.08)	**0.001**	1.05 (1.02, 1.08)	**0.002**	1.05 (1.02, 1.09)	**0.002**
P for log likelihood ratio test		**0.004**		**0.037**		**0.038**
DFS						
Age, years☆	1.01(0.99-1.02)	0.275	1.01(0.99-1.02)	0.180	1.01(0.99-1.03)	0.171
Inflection point★						
Age<41 years	0.92 (0.86, 0.97)	**0.004**	0.92 (0.87, 0.98)	**0.008**	0.92 (0.86, 0.98)	**0.006**
Age>41 years	1.02 (1.01, 1.04)	**0.010**	1.02 (1.00, 1.04)	**0.013**	1.02 (1.01, 1.04)	**0.007**
P for log likelihood ratio test		**0.004**		**0.007**		**0.004**

Model 1: unadjusted. Model 2: adjusted for tumor size, molecular subtype. Model 3: adjusted for tumor size, molecular subtype, lymph nodes in the axilla status, operation type, adjuvant therapy, hormonal therapy. *Cox proportional hazards models were used to estimate HRs and 95% CIs. ☆Fitting model by standard Cox proportional hazards model. ★Fitting model by two-piecewise Cox proportional hazards model.

The bold values denote statistical significance at P<0.05 level.

### Association of age with OS, BCSS and DFS

**Figure 3:**
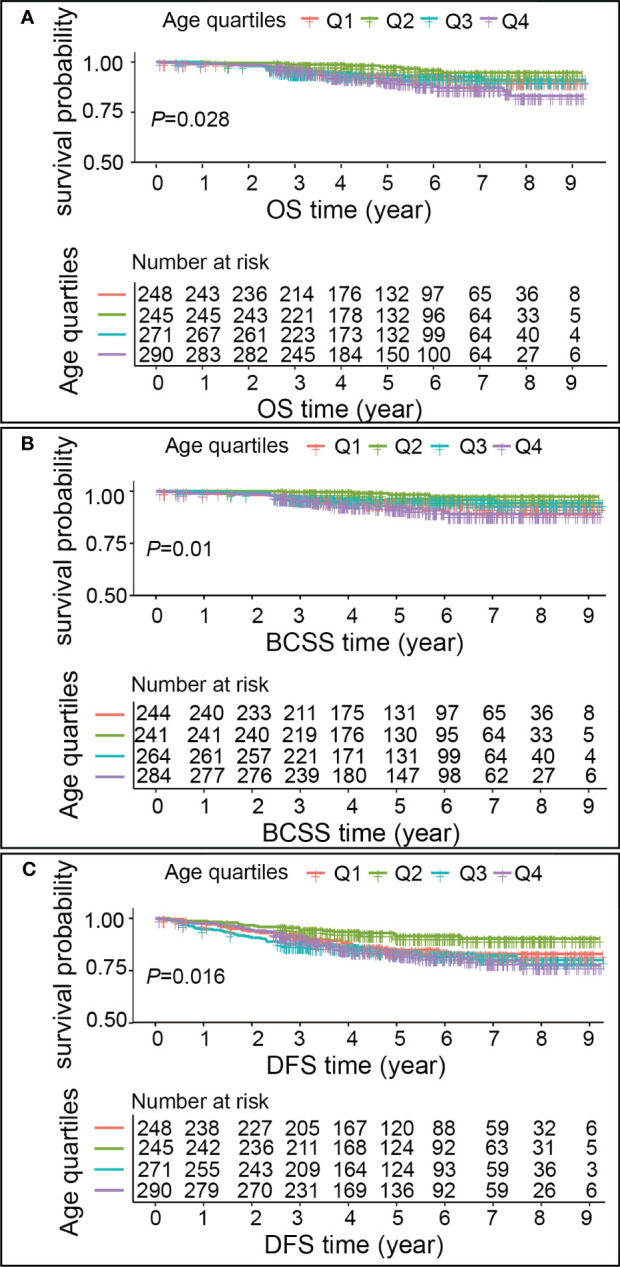
Kaplan–Meier estimates of OS, BCSS, DFS in different age groups. **(A)** OS, **(B)** BCSS, **(C)** DFS. Each graph comprises two components. The upper segment depicts the survival rates of breast cancer patients across various age groups, corresponding to the follow-up time. The horizontal axis represents the follow-up time, while the vertical axis denotes the survival probability. The lower segment of the graph illustrates the number of breast cancer patients in each age group who experienced a risk of survival at a specific follow-up time.

A median follow-up of 4.86 years after breast cancer diagnosis was observed, with an interquartile range of 3.26-6.83 years. Out of the total sample size, 71 patients (6.74%) experienced mortality, with 50 of those cases (70.42%) being attributed to breast cancer. Additionally, 144 patients (13.66%) encountered a recurrence of the condition. Kaplan–Meier outcomes showed that breast cancer patients age group in Q2 had a higher survival than in Q1, Q3 and Q4 patients (*P*<0.05) (**Figure 3**). The OS, BCSS, and DFS risks were higher in patients with Q1, Q3, and Q4 ages compared to Q2 (*P*<0.05). However, OS risk was non-significantly higher in Q1 compared with Q2 (*P*>0.05), and BCSS risk was non-significantly higher in Q3 compared with Q2 (*P*>0.05) (**Table 3**).

**Table 3:** Association between age and OS, SOS, and DFS among 1054 breast patients.

HR (95% CI)*	Age(years)			
	Q1(22-43years)	Q2(44-50years)	Q3(51-58years)	Q4 (59-90years)
OS				
Model 1	2.14 (0.92, 4.95)	1	2.18 (0.95, 5.01)	3.16 (1.44, 6.93)
Model 2	2.10 (0.90, 4.86)	1	2.13 (0.93, 4.90)	3.10 (1.41, 6.82)
Model 3	2.09 (0.90, 4.84)	1	2.44 (1.05, 5.65)	3.38 (1.51, 7.54)
BCSS				
Model 1	3.28 (1.07, 10.07)	1	2.66 (0.85, 8.35)	4.99 (1.72, 14.49)
Model 2	3.32 (1.08, 10.20)	1	2.58 (0.82, 8.10)	4.95 (1.70, 14.40)
Model 3	3.26 (1.06, 10.04)	1	2.88 (0.91, 9.11)	5.14 (1.74, 15.17)
DFS				
Model 1	1.88 (1.07, 3.28)	1	2.21 (1.29, 3.78)	2.22 (1.30, 3.78)
Model 2	1.89 (1.08, 3.30)	1	2.11 (1.23, 3.62)	2.20 (1.29, 3.75)
Model 3	1.90 (1.09, 3.33)	1	2.22 (1.29, 3.83)	2.29 (1.33, 3.94)

Model 1: unadjusted. Model 2: adjusted for tumor size, molecular subtype. Model 3: adjusted for tumor size, molecular subtype, LN positive, operation type, adjuvant therapy, hormonal therapy. *Cox proportional hazards models were used to estimate HRs and 95% CIs.

## Discussion

Extensive research has been conducted to investigate the correlation between age at diagnosis and survival rates in breast cancer (6, 10, 11, 16). Findings indicate that younger breast cancer patients are at a higher risk of developing malignancy and experiencing a less favorable prognosis (21, 22). Conversely, older women tend to exhibit indolent tumor characteristics (23), however, their prognosis is compromised by inadequate treatment or excessive intervention (24). Consequently, the prognostic implications of breast cancer remain a subject of controversy contingent upon the age at diagnosis.

In our study, we explored the relationship between diagnosis age and OS、BCSS and DFS. The findings revealed that breast cancer patients age group in Q2 had a higher survival than in Q1, Q3 and Q4 patients in our study.

Regarding OS, a recent study has revealed a significant improvement in the 10-year overall survival (OS) rates among breast cancer patients aged 40 years or older, as compared to those under the age of 40 (25). However, another study did not reveal a statistically significant difference in 5-year OS between the two age groups (26), which may be attributed to the shorter duration of follow-up. Wong et al. reported a U-shaped relationship between age at diagnosis and OS, with a nadir at 45 years (27). Our investigation also revealed a U-shaped association between diagnosis age and OS, with the lowest point at 44 years, consistent with Wong et al. findings. The study demonstrated a decline and subsequent rise in OS among breast cancer patients with advancing age. Additionally, there was a heightened susceptibility to competing background mortality following the lowest age, which exerted a greater impact on overall survival than breast cancer mortality alone. Notably, patients falling within the Q2 category exhibited superior OS compared to those in Q1, Q3, and Q4, with an age range of 44-50 years.

This finding aligns with the results conducted by Chen et al. which revealed that patients aged 40-49 years experienced significantly better OS than other patient groups (28). However, OS were non-significantly higher in Q1compared with Q2, which may be due to the short follow-up period in our study, resulting in a failure of statistical difference between the two.

For BCSS, several studies have suggested that younger age is associated with poorer BCSS in breast cancer patients (29–31). Wong et al. observed a non-linear relationship between age and BCSS, with the highest risk of breast cancer mortality observed in the youngest patients, followed by a linear decrease in hazard at a rate of 5% per year with increasing age (95% CI = 2%-8%; P = 0.001) (27). The risk of breast cancer mortality appears to remain low and relatively constant until approximately 50 years of age. Our investigation revealed a U-shaped relationship between age and BCSS. The study displayed that BCSS decreases with age until the age of diagnosis is 44 years and then shows an increase in breast cancer patients. The different findings of the two studies may be due to the fact that most patients with mastectomy in our study compared to Wong et al. study. Another study showed that patients aged 60-69 years had best BCSS among patients in other groups (28). However, our study findings indicate that individuals aged 44-50 years exhibited superior BCSS, while the BCSS in Q3 was insignificantly higher than that in Q2. It is worth considering that the limited sample size of our study, being conducted solely at a single center, may have influenced these results.

For DFS, the available literature indicates that younger breast cancer patients exhibit inferior DFS outcomes compared to their older counterparts (32, 33). Specifically, studies have demonstrated a statistically significant reduction in DFS among breast cancer patients under the age of 40 in comparison to those aged 40 years and above (25, 34). Furthermore, one study has identified a correlation between age and the formation of an L-shaped DFS curve (27). Notably, the evidence suggests that advancing age at diagnosis is associated with a decreased risk of breast cancer recurrence or mortality. The initial risk decreases rapidly with increasing age at presentation, suggesting that beyond approximately 40 years of age, the effect of age on breast cancer recurrence or death diminishes. In our study, however, age at diagnosis and DFS formed a U-shaped relationship. The initial risk decreases rapidly with increasing age at onset, with the curve showing the lowest value of this risk at approximately 41 years of age. this risk gradually increases with increasing age at onset after 41 years of age. And DFS rates was 13.66% (144 patients). Our result differed from Wong et al, possibly due to the inclusion of patients who were all breast-conserving patients in their study. Breast-conserving patients are generally younger, so an L-shape of age at diagnosis and DFS was observed. It May be due to different characteristics of the population of onset, too.

Our study showed that patients in the intermediate age (Q2) group had better survival compared to patients in the higher (Q3、Q4) and lower (Q1) age groups. Therefore, it suggests that we should not neglect the treatment of the last two. For lower age patients, the prognosis is poorer due to the more aggressive tumors such as TNBC diagnosed at a younger age of the tumor at the diagnosis time. And in the higher age patients, who often refuse standard treatment due to their poor health status, lower treatment acceptance or fear of poor tolerance to toxicity, such patients often do not receive standardized treatment leading to poor prognosis as well. For the higher age quartile in our study, patients had a significantly higher PR-negative tumors and mastectomy rate. In contrast, KI67 levels and adjuvant chemotherapy were significantly lower among the higher age quartile than among the lower quartile. One study showed that OS rates were not lower for young patients, despite their bigger tumors and poorer prognosis, results that patients may have benefitted from more aggressive treatment (35, 36). Additionally, another study has indicated that radiotherapy can improve OS and BCSS in women over 65 years of age with T3N0M0 (37). However, survival times did not differ between patients aged 18-45 years with and without radiotherapy. Therefore, for both groups of patients, clinical treatment plans should be individualized to improve their late survival.

Our study is based on data information from 1054 and is part of a large sample research center. However, it has several limitations. First, the median follow-up time in the study was only 4.86 years, which is not long enough. Second, the study used a retrospective data, which may have introduced sampling bias and incomplete information in case collection. for example, there was no geriatric assessment in our paper; missing histopathological characterization, TNM staging and molecular classification; the type of adjuvant treatment did not include the target treatment, no clear quantification of patients undergoing radiotherapy and chemotherapy; or no clarification of the percentage of patients with luminal tumors who received endocrine therap. Finally, it belongs to a monocentric study. These above may have unknown potential impact on our findings.

## Conclusions

Upon examination of the sample size contained within our research center’s database, it has been determined that the prognostic significance of age in relation to OS, BCSS, and DFS is subject to variation based on age. Our analysis has revealed a U-shaped correlation between age and breast cancer outcomes. These findings hold potential for serving as a foundation for future research endeavors focused on formulating personalized treatment strategies for patients across different age groups.

## Data availability statement

The raw data supporting the conclusions of this article will be made available by the authors, without undue reservation. Requests to access these datasets should be directed to Jianrong Yang, gandansurgery2014@163.com.

## Ethics statement

The studies involving humans were approved by The Ethics Committee of the Guangxi Zhuang Autonomous Region People’s Hospital (Ethics-KY-QT-202205). The studies were conducted in accordance with the local legislation and institutional requirements. Written informed consent for participation was not required from the participants or the participants’ legal guardians/next of kin in accordance with the national legislation and institutional requirements.

## Author contributions

YX: Writing – review & editing, Writing – original draft. YD: Writing – original draft, Data curation, Formal Analysis. SW: Data curation, Writing – review & editing. ZH: Writing – review & editing. LL: Supervision, Writing – review & editing. KH: Writing – review & editing. CW: Writing – review & editing. JX: Writing – review & editing. LD: Writing – review & editing. QHZ: Writing – review & editing. JZ: Writing – review & editing. QQZ: Methodology, Supervision, Writing – review & editing. JY: Data curation, Conceptualization, Methodology, Project administration, Writing – review & editing.

## References

[1] Sung H Ferlay J Siegel R Laversanne M Soerjomataram I Jemal A. Global cancer statistics 2020: GLOBOCAN estimates of incidence and mortality worldwide for 36 cancers in 185 countries. CA: Cancer J Clin (2021) 71(3):209–49. doi: 10.3322/caac.21660 33538338

[2] Extermann M Brain E Canin B Cherian M Cheung K de Glas N. Priorities for the global advancement of care for older adults with cancer: an update of the International Society of Geriatric Oncology Priorities Initiative. Lancet Oncol (2021) 22(1):e29–36. doi: 10.1016/S1470-2045(20)30473-3 33387502

[3] Chen W Zheng R Baade P Zhang S Zeng H Bray F. Cancer statistics in China, 2015. CA: Cancer J Clin (2016) 66(2):115–32. doi: 10.3322/caac.21338 26808342

[4] DeSantis C Ma J Gaudet M Newman L Miller K Goding Sauer A. Breast cancer statistics, 2019. CA: Cancer J Clin (2019) 69(6):438–51. doi: 10.3322/caac.21583 31577379

[5] Hankey B Miller B Curtis R Kosary C. Trends in breast cancer in younger women in contrast to older women. J Natl Cancer Institute Monogr (1994) 16):7–14. 7999473

[6] Xie Y Gou Q Zhang Y Xie K Zheng D Luo C. Association between age at initial diagnosis and post-metastasis mortality among women with recurrent metastatic breast cancer in China. BMC Cancer (2022) 22(1):385. doi: 10.1186/s12885-022-09454-y 35397518 PMC8994897

[7] Ibrahim A Salem M Hassan R. Outcome of young age at diagnosis of breast cancer in South Egypt. Gulf J Oncol (2014) 1(15):76–83. 24610292

[8] Alieldin N Abo-Elazm O Bilal D Salem S Gouda E Elmongy M. Age at diagnosis in women with non-metastatic breast cancer: Is it related to prognosis? J Egyptian Natl Cancer Institute (2014) 26(1):23–30. doi: 10.1016/j.jnci.2013.08.005 24565679

[9] Grybach S Polishchuk L Chekhun V. Analysis of the survival of patients with breast cancer depending on age, molecular subtype of tumor and metabolic syndrome. Exp Oncol (2018) 40(3):243–8. doi: 10.31768/2312-8852.2018.40(3):243-248 30285006

[10] van de Water W Markopoulos C van de Velde C Seynaeve C Hasenburg A Rea D. Association between age at diagnosis and disease-specific mortality among postmenopausal women with hormone receptor-positive breast cancer. JAMA (2012) 307(6):590–7. doi: 10.1001/jama.2012.84 22318280

[11] Ojala K Meretoja T Mattson J Leidenius M. Surgical treatment and prognosis of breast cancer in elderly - A population-based study. Eur J Surg Oncol (2019) 45(6):956–62. doi: 10.1016/j.ejso.2019.01.019 30691722

[12] Billena C Wilgucki M Flynn J Modlin L Tadros A Razavi P. 10-year breast cancer outcomes in women ≤35 years of age. Int J Radiat oncology biology Phys (2021) 109(4):1007–18. doi: 10.1016/j.ijrobp.2020.10.022 PMC8006530 33371964

[13] Wei X Li X Xin X Tong Z Zhang S. Clinical features and survival analysis of very young (age<35) breast cancer patients. Asian Pacific J Cancer Prev APJCP (2013) 14(10):5949–52. doi: 10.7314/APJCP.2013.14.10.5949 24289606

[14] Han W Kang S. Relationship between age at diagnosis and outcome of premenopausal breast cancer: age less than 35 years is a reasonable cut-off for defining young age-onset breast cancer. Breast Cancer Res Treat (2010) 119(1):193–200. doi: 10.1007/s10549-009-0388-z 19350387

[15] Zhong W Tan L Jiang W Chen K You N Sanders A. Effect of younger age on survival outcomes in T1N0M0 breast cancer: A propensity score matching analysis. J Surg Oncol (2019) 119(8):1039–46. doi: 10.1002/jso.25457 30892719

[16] Szollár A Újhelyi M Polgár C Oláh E Pukancsik D Rubovszky G. A long-term retrospective comparative study of the oncological outcomes of 598 very young (≤35 years) and young (36-45 years) breast cancer patients. Eur J Surg Oncol (2019) 45(11):2009–15. doi: 10.1016/j.ejso.2019.06.007 31189512

[17] Feng F Wei Y Zheng K Li Y Zhang L Wang T. Comparison of epidemiological features, clinicopathological features, and treatments between premenopausal and postmenopausal female breast cancer patients in western China: a retrospective multicenter study of 15,389 female patients. Cancer Med (2018) 7(6):2753–63. doi: 10.1002/cam4.1503 PMC6010855 29673111

[18] Wang K Ren Y Li H Zheng K Jiang J Zou T. Comparison of clinicopathological features and treatments between young (≤40 years) and older (>40 years) female breast cancer patients in west China: A retrospective, epidemiological, multicenter, case only study. PloS One (2016) 11(3):e0152312. doi: 10.1371/journal.pone.0152312 27031236 PMC4816508

[19] Jiang Z Song E Wang X Wang H Wang X Wu J. Guidelines of chinese society of clinical oncology (CSCO) on diagnosis and treatment of breast cancer (2020 version). Trans Breast Cancer Res (2020) 1(October):1–25. doi: 10.21037/TBCR-2020-2

[20] Guideline for HER2 detection in breast cancer, the 2019 version. Zhonghua bing li xue za zhi = Chin J Pathol (2019) 48(3):169–75. doi: 10.3760/cma.j.issn.0529-5807.2019.03.001 30831640

[21] Derks M Bastiaannet E van de Water W de Glas N Seynaeve C Putter H. Impact of age on breast cancer mortality and competing causes of death at 10 years follow-up in the adjuvant TEAM trial. Eur J Cancer (Oxford Engl 1990) (2018) 99:1–8. doi: 10.1016/j.ejca.2018.04.009 29885375

[22] Bouferraa Y Haibe Y Chedid A Jabra E Charafeddine M Temraz S. The impact of young age (< 40 years) on the outcome of a cohort of patients with primary non-metastatic breast cancer: analysis of 10-year survival of a prospective study. BMC Cancer (2022) 22(1):27. doi: 10.1186/s12885-021-09100-z 34980002 PMC8722326

[23] Johnson R Anders C Litton J Ruddy K Bleyer A. Breast cancer in adolescents and young adults. Pediatr Blood Cancer (2018) 65(12):e27397. doi: 10.1002/pbc.27397 30156052 PMC6192832

[24] Di Lascio S Tognazzo E Bigiotti S Bonollo M Costa A Pagani O. Breast cancer in the oldest old (≥ 89 years): Tumor characteristics, treatment choices, clinical outcomes and literature review. Eur J Surg Oncol (2021) 47(4):796–803. doi: 10.1016/j.ejso.2020.10.008 33097334

[25] Kim H Kim S Freedman R Partridge A. The impact of young age at diagnosis (age <40 years) on prognosis varies by breast cancer subtype: A U. S. SEER Database analysis. Breast (Edinburgh Scotland) (2022) 61:77–83. doi: 10.1016/j.breast.2021.12.006 34923225 PMC8693310

[26] Walsh S Zabor E Flynn J Stempel M Morrow M Gemignani M. Breast cancer in young black women. Br J Surg (2020) 107(6):677–86. doi: 10.1002/bjs.11401 PMC7422000 31981221

[27] Wong F Tham W Nei W Lim C Miao H. Age exerts a continuous effect in the outcomes of Asian breast cancer patients treated with breast-conserving therapy. Cancer Commun (London England) (2018) 38(1):39. doi: 10.1186/s40880-018-0310-3 PMC6020242 29941044

[28] Chen H Zhou M Tian W Meng K He H. Effect of age on breast cancer patient prognoses: A population-based study using the SEER 18 database. PloS One (2016) 11(10):e0165409. doi: 10.1371/journal.pone.0165409 27798652 PMC5087840

[29] Fredholm H Magnusson K Lindström L Garmo H Fält S Lindman H. Long-term outcome in young women with breast cancer: a population-based study. Breast Cancer Res Treat (2016) 160(1):131–43. doi: 10.1007/s10549-016-3983-9 PMC5050247 27624330

[30] Dai D Zhong Y Wang Z Yousafzai N Jin H Wang X. The prognostic impact of age in different molecular subtypes of breast cancer: a population-based study. PeerJ (2019) 7:e7252. doi: 10.7717/peerj.7252 31309004 PMC6612417

[31] Sun H Huang W Ji F Pan Y Yang L. Comparisons of metastatic patterns, survival outcomes and tumor immune microenvironment between young and non-young breast cancer patients. Front Cell Dev Biol (2022) 10:923371. doi: 10.3389/fcell.2022.923371 35912097 PMC9329535

[32] Lian W Fu F Lin Y Lu M Chen B Yang P. The impact of young age for prognosis by subtype in women with early breast cancer. Sci Rep (2017) 7(1):11625. doi: 10.1038/s41598-017-10414-x 28912475 PMC5599495

[33] Cvetanovic A Popovic L Filipovic S Trifunovic J Zivkovic N Matovina-Brko G. Young age and pathological features predict breast cancer outcome - report from a dual Institution experience in Serbia. J BUON (2015) 20(6):1407–13. 26854434

[34] El Chediak A Alameddine R Hakim A Hilal L Abdel Massih S Hamieh L. Younger age is an independent predictor of worse prognosis among Lebanese nonmetastatic breast cancer patients: analysis of a prospective cohort. Breast Cancer (Dove Med Press) (2017) 9:407–14. doi: 10.2147/BCTT.S130273 PMC5479304 28670139

[35] Foo C Su D Chong C Chng H Tay K Low S. Breast cancer in young Asian women: study on survival. ANZ J Surg (2005) 75(7):566–72. doi: 10.1111/j.1445-2197.2005.03431.x 15972049

[36] Abdel-Razeq H Iweir S Abdel-Razeq R Rahman F Almasri H Bater R. Differences in clinicopathological characteristics, treatment, and survival outcomes between older and younger breast cancer patients. Sci Rep (2021) 11(1):14340. doi: 10.1038/s41598-021-93676-w 34253800 PMC8275803

[37] He M Lu X Gou Z. Effects of postmastectomy radiotherapy on survival in different age groups for patients with T3N0M0 breast cancer. Breast (Edinburgh Scotland) (2021) 60:247–54. doi: 10.1016/j.breast.2021.11.006 PMC8606543 34808436

